# Examining the Acute Effects of Classroom-Based Physical Activity Breaks on Executive Functioning in 11- to 14-Year-Old Children: Single and Additive Moderation Effects of Physical Fitness

**DOI:** 10.3389/fped.2021.688251

**Published:** 2021-08-03

**Authors:** Jeffrey D. Graham, Emily Bremer, Barbara Fenesi, John Cairney

**Affiliations:** ^1^Faculty of Health Sciences, Ontario Tech University, Oshawa, ON, Canada; ^2^Faculty of Kinesiology & Physical Education, University of Toronto, Toronto, ON, Canada; ^3^Faculty of Education, Western University, London, ON, Canada; ^4^School of Human Movement and Nutrition Sciences, The University of Queensland, Brisbane, QLD, Australia

**Keywords:** cognition, youth, exercise, school, mood, motivation, self-efficacy

## Abstract

**Objective:** Research supports the efficacy of acute, classroom-based, physical activity breaks on executive functioning in children. However, research pertaining to the effect of physical fitness on the acute physical activity—executive functioning relationship remains limited. The primary purpose of this study was to investigate the acute effects of classroom-based, teacher-delivered, physical activity breaks on executive functioning in 11–14-year-old children. We also investigated the potential moderating effects of both aerobic and musculoskeletal fitness on the acute physical activity—executive functioning relationship.

**Method:** Participants (*N* = 116) completed pre- and post-test assessments of executive functioning (i.e., inhibition, switching, and updating) separated by a classroom-based physical activity break or sedentary classroom work. We manipulated the dose (i.e., length) and type of physical activity breaks. With regards to dose, participants in the experimental conditions engaged in 5-, 10-, or 20-min of physical activity whereas controls completed sedentary classroom math work at their desk. With regards to type, one experimental condition completed traditional physical activity breaks whereas the other experimental condition completed academic physical activity breaks (i.e., performed mental math and physical activity). Participants' mood, motivation, and self-efficacy were also assessed following the experimental manipulations.

**Results:** Overall, executive function scores improved across each assessment following the physical activity breaks when compared to sedentary classroom work regardless of dose and type. Participants also reported more positive mood states, higher motivation to complete the executive function tests, and higher self-efficacy to perform the executive functions tests following the physical activity breaks. Single moderation analyses showed that low-moderate levels of aerobic fitness moderated the acute physical activity—executive functioning relationship. Additive moderation analysis showed, collectively, that both aerobic and musculoskeletal fitness moderated the acute physical activity—executive functioning relationship.

**Conclusion:** Findings from the present study provide evidence for the acute effects of short (i.e., 5–20 min) classroom-based physical activity breaks on executive functioning and psychological states in children. Results also suggest levels of both aerobic and musculoskeletal fitness moderate these effects, however future research is needed to further elucidate this complex relationship.

## Introduction

Strong evidence supports the association between regular participation in physical activity and various cognitive, mental, and physical health outcomes among children and adolescents (1, 2). Despite this evidence and various physical activity initiatives across many sectors of society, the majority of children and adolescents worldwide are not engaging in sufficient levels of physical activity to reap these known health benefits (3–5). Given children spend most of their day in a school setting, many researchers and policy makers have recently devoted a substantial amount of time and resources to understand how the school setting may be used as a means to improve children's overall health through increased participation in physical activity during school hours [for an illustrative review see (6)]. Indeed, emerging evidence provides support for the efficacy of physical activity interventions in school settings to improve various adaptive cognitive, physical, and academic outcomes (7, 8).

### Acute Exercise, Executive Functioning, and Classroom-Based Physical Activity Breaks

The school setting has also been targeted due to the evidence supporting the carryover effects of engaging in shorts bouts of physical activity and exercise on aspects of executive functions and cognition (9, 10). Executive functions refer to an array of higher order brain-based, or mental, processes that enable individuals to exert self-control and self-regulation over their behavior (11, 12). Executive functions can be divided into three core processes including “inhibition” which refers to the ability to suppress (or resist) automatic responses such as urges and distractions, “shifting” which refers to the ability to switch one's attention back and forth between multiple rules, mental sets, or tasks, and “updating” which refers to the maintenance of relevant information in working memory and the ability to process that information further. In turn, executive functions are related to many adaptive health outcomes across the life span (13, 14), including regular participation in physical activity (15, 16) as well as academic achievement and learning across a range of subjects (17–20). With regards to the acute effects of physical activity on executive functioning, ample research suggests relatively short bouts (e.g., a single 10–20-min bout) of various forms of physical activity and exercise (e.g., jogging, cycling, and circuit-based activities) can lead to short-term improvements on measures of executive functions (9, 10).

Given the understanding of the impact of acute physical activity on executive functioning, it is not surprising that many researchers have investigated ways to implement physical activity breaks within the classroom setting to not only improve learning and academic performance, but in some cases to increase enjoyment of the subject material and/or the learning environment (7, 19, 21). In these studies, researchers typically lead students through short bouts of various physical activities that can be performed behind a student's desk or as a group in an open area of the classroom. These activities often include aerobic or resistance-based physical activities that can be performed “on the spot” such as jumping jacks, burpees, push-ups, squats, split jumps, and jogging in place (among others). Prior to and following the acute bout of physical activity children are assessed one-on-one, or as a group, on various measures of executive functioning and academic performance (e.g., math). When compared to sedentary control conditions (e.g., regular classroom work), students who engaged in the physical activity break(s) generally show improvements on measures of executive functioning and academic performance both acutely and over time (7, 19, 21). Yet, one limitation to this area of research is that researchers are often the ones implementing the physical activity breaks with very few studies utilizing the teacher as the sole leader of the breaks. This has implications for both the ecological validity of the designs, but also pragmatic concerns over adoption and feasibility of implementation.

Besides the need to increase ecological validity and translate research into practice, it is important to acknowledge that various physical activity initiatives within school settings are not being adhered to at recommended levels. For instance, in Ontario, Canada, the Ministry of Education released a DPA policy in 2005 which mandated that all publicly funded elementary schools provide at least 20 min of sustained moderate-vigorous physical activity to their students each day during instructional time (22). However, since the inception of this mandate, adherence has been very poor (23, 24) with research showing that <50% of students were actually provided with an opportunity to engage in DPA (25). While teachers recognize the value of DPA, they often struggle to implement 20 min of sustained DPA (23, 25). The Comprehensive School Physical Activity Program (CSPAP) model (26), and its iterations [for a review see (6)], stresses the need to not only share information and resources with teachers about DPA but also to provide training opportunities and on-going support during the initial stages of DPA implementation throughout the school day and especially within the classroom setting [also see (27)].

The implementation of physical activity breaks within the classroom setting has also sparked an emerging area of research which incorporates academic content (e.g., mathematics, language, and geography) alongside physical activity (7, 19, 21). For example, this could include presenting children with mental math problems (e.g., 5 × 5 = ?) whereby they are asked to write down the answer to the problem and then perform that many repetitions of a certain physical activity (e.g., 25 jumping jacks). These academic physical activity breaks are not only practical as they can preserve teaching time while also reaping the acute benefits of physical activity, but the efficacy for these breaks is also supported through a combination of neuroscientific, developmental, and embodied cognition perspectives [for reviews see (19, 28, 29)]. In short, these reviews and others (30) have proposed various intriguing reasons for why certain types of physical activity that require a high degree of cognitive engagement may be more beneficial than traditional forms of physical activity due to the unique connection between brain regions governing cognition (e.g., prefrontal cortex) and movement (e.g., cerebellum). For instance, these brain regions are fundamentally interconnected such that they co-activate when performing tasks primarily requiring cognition (e.g., math and reading) or motor behavior (e.g., running and balancing) and together support successful execution and performance on both types of tasks (31–34). In turn, academic physical activity breaks may not only pre-activate the same brains regions and cognitive processes needed for subsequent academic material (35, 36) but they may also strengthen the connection between these regions over time and support performance on related tasks (37). Although research in this area is still emerging, reviews and meta-analyses of intervention studies in children suggest the effects of cognitively engaging physical activity on aspects of executive functioning and cognition are superior to more traditional forms of physical activity (38–41). In addition, acute academic physical activity breaks have been shown to be superior to traditional forms of active and/or sedentary classroom learning environments (19), however the number of studies in this area remains limited.

While the above supports the efficacy for physical activity requiring an enhanced degree of cognitive engagement (i.e., academic physical activity or cognitively engaging physical activity) on aspects of executive functioning and cognition in general, and when compared to more traditional forms of physical activity, it is important to acknowledge and discuss a key difference between these two types of activity. Specifically, *academic physical activity* incorporates academic material alongside physical activity whereas *cognitively engaging (or challenging) physical activity* increases the concurrent cognitive demands of performing the physical activity itself [for a review see (30)]. For example, this could include juggling while jogging or playing a team sport (e.g., basketball) that has additional rules. Although academic physical activity is essentially a subtype of cognitively engaging physical activity, research suggests academic physical activity breaks can facilitate learning to a greater extent when compared to other types of cognitively engaging activity breaks and traditional activity breaks [for a comprehensive review and conceptual model see (19)].

Despite the increasing evidence for traditional, academic, and cognitively engaging physical activity breaks on aspects of executive functioning and cognition, there is no consensus for the optimal length of these acute physical activity breaks. This is not entirely surprising given various findings within the broader literature examining the acute effects of physical activity or exercise on executive functioning. For instance, a meta-analysis by Chang et al. (42) suggested that acute bouts lasting 11-min or longer generally resulted in improvements in executive functioning and cognition. However, as pointed out by Pontifex et al. (10), this conclusion may be partially attributable to the characteristics of the studies included in their analysis and the number conducted up to that point in time. Indeed, the updated review by Pontifex et al. (10) found the majority of studies within the extant literature have utilized acute bouts lasting 16–35 min (88% of the literature) while much less have utilized bouts lasting 10-min or less. In turn, as suggested by Pontifex et al. (10), an 11-min threshold does not negate the fact that shorter durations may also be effective given the array of possible considerations (e.g., population, age, type of activity, length of time between activity bout and cognitive assessment, etc.). Although there are many other factors to consider besides duration (e.g., intensity), it is important to acknowledge that the classroom environment may suit a range of durations (e.g., 5, 10, or 20 min) depending on the subject, grade level, or lesson plan for that day while the intensity may largely be held constant due to the nature of the activities that can be performed in a classroom setting. As such, shorter bouts may be more feasible to implement within the classroom and evidence supports comparable effects between an acute 10- vs. a 20-min classroom physical activity break on math scores (43). Other research has provided support for acute academic physical activity breaks and cognitively engaging activity breaks lasting 10-min in length on aspects of executive functioning and enjoyment within the classroom (36, 44). While the evidence for acute physical activity breaks lasting <10-min remains limited, intervention research suggests traditional physical activity breaks lasting 4-min (45) and both traditional and academic physical activity breaks lasting 5-min (46) can improve on-task behavior and math performance over time. Thus, emerging evidence supports the efficacy for the acute effects of short duration classroom-based physical activity across a variety of adaptive outcomes, however the acute effects for activity breaks lasting <10-min remains scarce and requires additional research.

### Physical Fitness, Executive Functioning, and Acute Exercise

Similar to physical activity, aspects of physical fitness are also intricately linked to overall health, executive functioning, and academic achievement (47–51). For instance, children and youth who have higher levels of aerobic fitness are generally healthier and perform better on tests of executive functioning and academic achievement. While the majority of the literature has focused on aspects of aerobic fitness, emerging evidence suggests aspects of musculoskeletal fitness are also related to executive functioning and academic achievement among children and youth (49, 52–54). Understanding the potential influence that both types of fitness may have on executive functioning, both independently and collectively, is important as recent research suggests high levels of both types of fitness may enact an added or combined benefit on executive functioning (53).

Given the relationship between high levels of physical fitness and executive functioning in general, it is plausible that the effects of acute exercise on executive functioning may also be affected (i.e., moderated) by levels of physical fitness. Despite this intriguing assumption, the evidence remains mixed [for meta-analyses see (42, 55)]. For instance, experimental research has shown that individuals with higher levels of aerobic fitness benefit the most on measures of executive functioning following acute exercise (56, 57) whereas other research showed that those with moderate levels of aerobic fitness benefited the most (58). Yet, a sub-analysis from a meta-analytic review showed no effects for aerobic fitness on the acute exercise—executive functioning relationship (55) indicating that acute exercise uniformly, and positively, affected executive functioning across varying levels of physical fitness. However, these findings were strictly limited to acute aerobic exercise performed at a moderate intensity. More recent experimental evidence continues to remain mixed across aerobic fitness levels, exercise intensities, age groups, and measures of executive functioning [i.e., (59–62)] suggesting future research is warranted to further elucidate this complex relationship.

There are a couple other notable limitations to previous research investigating the potential moderating effects of physical fitness on the acute exercise—executive functioning relationship. First and foremost, as far as we are aware, previous research has solely investigated aspects of aerobic fitness and therefore the potential moderating effects of musculoskeletal fitness remain unknown. In addition, the majority of previous studies have created groups that represented varying levels of fitness (e.g., low, moderate, and high) based on standardized guidelines (e.g., American College of Sports Medicine guidelines) or normative data. While this method is entirely justified in order to investigate different fitness levels based on pre-determined standards or guidelines, it also has certain shortcomings when examining potential moderating effects. For instance, these include various statistical limitations and criticisms against conducting a moderation analysis through the use of groups as the moderator rather than a continuous variable [see (63), *Artificial Categorization and Subgroups Analysis*, p. 263–265]. It is also plausible that there may be a certain point along the fitness continuum where physical fitness may become a meaningful and significant contributor to executive functioning performance following acute exercise. This point, however, can only be identified through probing an interaction of a moderation analysis that used a continuous variable as the moderator. Based on the sample (e.g., children, adolescents, or older adults) and measure of fitness (e.g., graded exercise test, shuttle run, or standing long jump) it may be important to know when fitness becomes a significant predictor and what the relationship may look like following that point (e.g., is the relationship linear or curvilinear) for that specific measure of fitness. In other words, if performance on the shuttle run test is a moderator of the acute exercise—executive functioning relationship among children aged 11–12 years, it would be interesting to know the specific stage or number of laps that was achieved to reap the acute effects of exercise on executive functioning. Within a school setting, this information could be utilized by school boards and physical education specialists whereby this aspect of fitness may be targeted and trained in a similar sample of children in order to reap the benefits of acute classroom-based physical activity breaks on aspects of executive functioning, learning, and academic performance.

**Figure 1:**
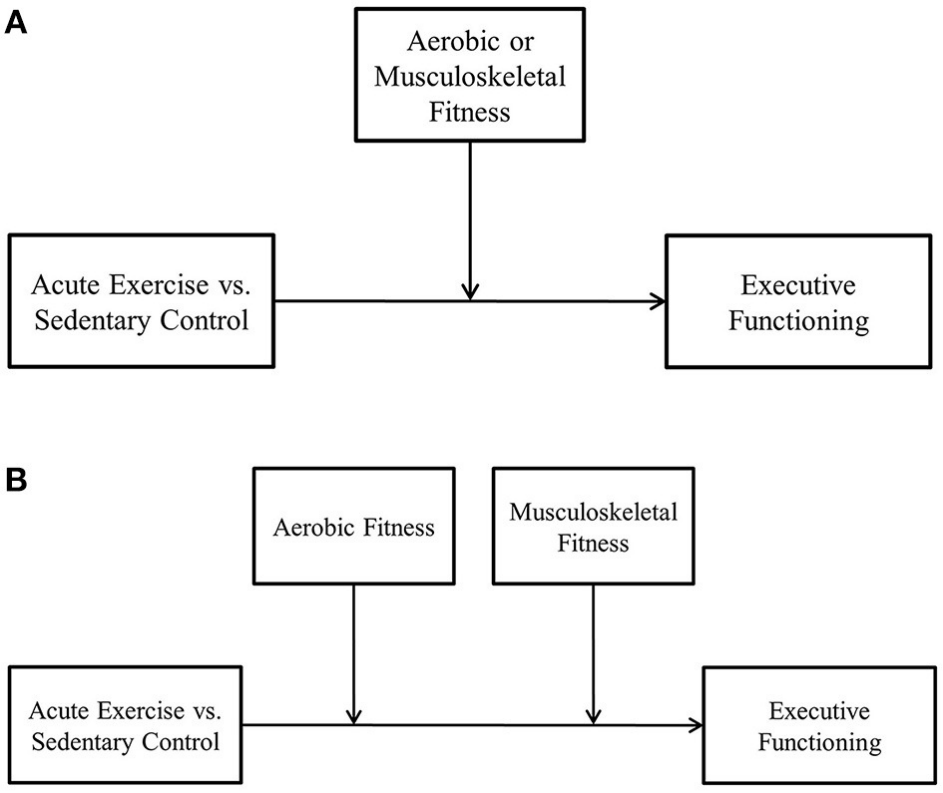
**(A)** Acute exercise—executive functioning single moderation example. **(B)** Acute exercise—executive functioning additive moderation example.

Furthermore, as recent cross-sectional research suggests both high levels of aerobic and musculoskeletal fitness may provide a combined benefit on executive functioning (53), it is also plausible that both types of fitness may provide a combined effect on executive functioning following acute exercise. Extending the traditional single moderation model (depicted in Figure 1A), additive moderation analysis (depicted in Figure 1B) provides researchers with the ability to examine the potential combined effect of both types and levels of fitness on the acute exercise—executive functioning relationship within a single analysis.

### Purpose and Hypotheses

The primary objective of this study was to investigate the acute effects of a classroom-based, teacher-led, physical activity break on executive functioning in 11–14-year-old children when compared to sedentary classroom work. To maintain a high degree of ecological validity, the study was conducted within a school setting during math class. In addition, we manipulated the dose (or length) of the physical activity breaks as well as the type of physical activity breaks. With regards to dose, children in the experimental conditions completed a 5-, 10-, or 20-min physical activity break whereas controls completed sedentary classroom math work at their desk during this time. With regards to type, one experimental condition completed traditional physical activity breaks whereas the other experimental condition completed academic physical activity breaks. Consistent with the literature reviewed above, we hypothesized that an acute classroom-based physical activity break would lead to improvements in executive functioning when compared to sedentary classroom work regardless of the dose. We also hypothesized that academic physical activity breaks would lead to a greater change in executive functioning when compared to traditional physical activity breaks and the control condition, again regardless of the dose. We chose not to formulate hypotheses relating to dose due to discrepancies within the extant literature regarding the limited number of studies conducted utilizing bouts lasting <10-min (10), the fact that few studies have directly compared the effects of varying durations within a single study [e.g., (43)], and emerging evidence suggesting acute academic physical activity bouts lasting <10-min can lead to improvements in aspects of executive functioning and academic performance (19). Therefore, our analyses with regards to dose were considered exploratory.

A secondary objective was to investigate the potential moderating effects of physical fitness (i.e., both aerobic and musculoskeletal fitness) on the acute physical activity—executive functioning relationship. Given inconsistencies and limitations within the literature discussed above, we explored the potential independent and combined moderating effects of both aerobic and musculoskeletal fitness through the use of a continuous variable as the moderator and by conducting separate single moderation analyses (see Figure 1A) and through an additive moderation analysis (see Figure 1B).

## Methods

### Participants and Design

Participants included 11- to 14-year-old children in grades 6–8 (*N* = 116; *n* = 58 girls, *M*_*age*_ = 12.19 ± 0.93) who were part of a larger 6-week intervention study investigating the effects of classroom-based, teacher-led, physical activity breaks on aspects of physical fitness, executive functioning, and psychosocial well-being. The larger study was completed in partnership with the Hamilton-Wentworth Catholic District School Board and utilized a 3 (activity type: traditional physical activity break vs. academic physical activity break vs. sedentary math) × 3 (activity dose: 4 × 5-min vs. 2 × 10-min vs. 1 × 20-min) × 2 (time: pre-intervention vs. post-intervention) between-subjects experimental design. Three schools were chosen to participate by the Hamilton-Wentworth Catholic District School Board and each were assigned an activity dose by the school board so that all of the students in the experimental classes within each school participated in the same dosage of physical activity. The physical activity breaks were delivered daily by the teachers, in the classroom, during a 75-min math class period and the doses were delivered so that physical activity was equally spread throughout the class period. For example, the 2 × 10-min group completed ~18-min of math, engaged in 10-min of physical activity, completed another 18-min of math, then engaged in 10-min of physical activity, and finally 18-min of math. Whereas, the 4 × 5-min group had four equally spaced interval physical activity breaks throughout math class and the 20-min group only had one 20-min break in the middle of math class.

All of the grade 2–8 students and teachers from each school were invited to participate in the larger study. Students who were not enrolled in the longitudinal study also participated in the daily physical activity breaks alongside their peers who consented to participate in the study. In addition, only students in grades 6–8 who were apart of the larger study were also invited to participate in the acute portion of the study (presented in this paper) due to feasibility issues and in order to minimize class distractions among the younger students as per recommendations from the school board. Teachers who agreed to participate in one of the physical activity conditions were provided with the option to choose either the traditional physical activity break condition or the academic physical activity break condition. If teachers had no preference between the two physical activity conditions, they were then randomly assigned by the researchers to one of the two conditions at the grade level. For instance, if two grade 6 teachers at one school agreed to participate and had no preference, they were randomized so that one teacher delivered traditional physical activity breaks and the other delivered academic physical activity breaks. The study was approved by the McMaster University Research Ethics Board and the Hamilton-Wentworth Catholic District School Board Research Ethics Committee. Parents provided informed written consent and students provided informed written assent before participation in the study.

The present study is an examination of the acute effects of a single physical activity break, compared to sedentary math work, on executive functioning and the potential moderating effect of physical fitness. A randomization schedule was generated (random.org) for grade 6–8 students who agreed to participate in the acute portion of the study. However, due to unforeseen limited access to students from one school, we ended up with unequal group sizes across activity type and dose. That is, our final sample (*N* = 116) included 67 sedentary control participants, 37 academic physical activity break participants, and 12 traditional physical activity break participants. Due to the low number of participants in the traditional physical activity break condition, we combined the participants in the physical activity break conditions to form one condition. Specifically, this study utilized a 2 (activity type: physical activity break vs. sedentary math) × 3 (activity dose: 1 × 5-min vs. 2 × 10-min vs. 1 × 20-min) × 2 (time: pre-manipulation vs. post-manipulation) between-subjects experimental design, which included: a 5-min physical activity break condition (*n* = 19), a 10-min physical activity break condition (*n* = 10), a 20-min physical activity break condition (*n* = 20), a 5-min sedentary math condition (*n* = 23), a 10-min sedentary math condition (*n* = 23), and a 20-min sedentary math condition (*n* = 21).

### Procedure

The data was collected over the first 3-week period of the intervention, during math class, within regular school hours (i.e., 8:30 a.m.−3:00 p.m.). Participants were accompanied by a trained research assistant to a quiet room within the school to complete the pre-manipulation study measures. They were first fitted with a heart rate monitor, then completed measures of mood and motivation (see section Secondary Outcome Measures below), followed by the pre-test executive function assessments (see section Primary Outcome Measures below). They were then walked back to their classroom by the research assistant and were exposed to their respective experimental manipulation. Children in the physical activity break conditions participated in a 5-, 10-, or 20-min, teacher-delivered, physical activity break with their fellow classmates. Whereas, participants in the sedentary control condition resumed their regular classroom math work for their assigned dose manipulation (i.e., 5-, 10-, or 20-min of math work).

Heart rate was measured continuously during the experimental manipulations using a Polar H7 chest strap that was synced to an iPad using the Polar GoFit application (Polar Canada, Lachine, Quebec). Exercise intensity was monitored through the Polar GoFit application which was displayed at the front of the classroom on a smartboard. Each student had their own unique identifier box (displayed on a smartboard) and exercise intensity was synced through their heart rate monitor. Exercise intensity was set to 60–80% of the participants' maximum heart rate (HRmax) which was predetermined based off their age (i.e., 220-age = HRmax) and was chosen as it is commonly used when examining the acute effects of physical activity or exercise on executive functioning (10, 64). The participants' unique identifier box would change colors based on their heart rate in relation to the predetermined intensity, with gray indicating too low of an intensity, blue (60–70% of HRmax) and green (70–80% of HRmax) indicating the correct intensities, and yellow (80–90% of HRmax) and red (90–100% of HRmax) indicating too high of an intensity. Participants were encouraged to self-regulate their own intensity throughout the physical activity break so that they kept their unique identifier box in the blue or green intensity zones, however we did not extract heart rate data for this study.

**Figure 2:**
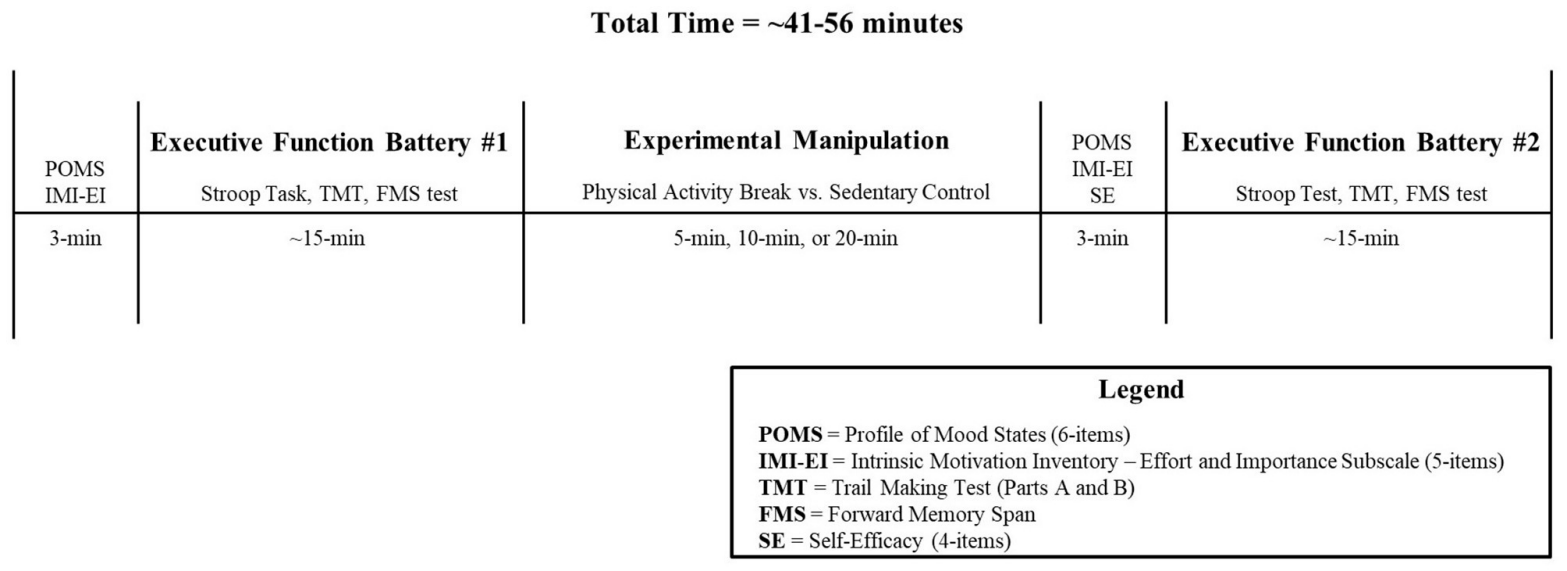
Experimental protocol.

Following the experimental manipulation, the research assistant walked the participant back to the same quiet room to complete the post-manipulation measures. That is, participants first completed the task self-efficacy measure (see section Secondary Outcome Measures below), followed by the mood and motivation measures, and then the post-test executive function assessments. Upon completion of the post-test executive function assessments, participants were provided with a $15 Indigo gift card. See Figure 2 for an overview of the experimental protocol.

### Experimental Manipulations

#### Activity Type

The physical activity break manipulations were teacher-delivered and occurred during math class. Prior to the study, and in line with expert recommendations (6, 27), our research group shared information with teachers on the acute and long-term benefits of classroom-based physical activity breaks and strategies to effectively deliver these breaks, provided all the necessary intervention materials, and held training sessions with regards to the effective delivery of the physical activity breaks. We also checked in daily to provide additional support, guidance, and to troubleshoot any problems with implementation.

##### Traditional Physical Activity Break

The traditional physical activity breaks included tasks suitable to be performed standing behind a desk or in a small open space within the classroom. The physical activities were delivered through the Ontario Physical and Health Education Association's (OPHEA) *50 Fitness Activity* cards (https://teachingtools.ophea.net/sites/default/files/pdf/pdm_50fitnessactivities_17se19.pdf). Examples of activities included variations of jumping jacks, running on the spot, lunges, and tuck jumps, among others. The teachers were also provided with cards that had numbers from 1 to 50 printed on them. Teachers delivered the physical activity break by randomly selecting a number (i.e., 10) alongside randomly selecting an activity card (i.e., tuck jumps), and then students would perform that many repetitions of that activity (i.e., 10 tuck jumps). This process would be repeated for the entire duration of the physical activity break. In addition, teachers were encouraged to use number cards ranging from 5 to 20 to maintain a moderate-vigorous physical activity intensity and to be feasible for students to accomplish. However, teachers were also encouraged to adapt their physical activity breaks to the physical ability of the class and could use a larger (i.e., 5–35) or smaller (i.e., 5–15) range of numbers.

##### Academic Physical Activity Break

The academic physical activity breaks were identical to the traditional physical activity breaks with the exception that teachers were provided with grade-appropriate math problem flash cards in place of the number cards. In other words, teachers would randomly select a math problem card (i.e., 5 × 5 = ?) and then randomly select an activity card (i.e., tuck jumps). Students would then perform mental math, write the answer down on a piece of paper, and perform that many repetitions (i.e., 25 tuck jumps). Again, teachers were encouraged to modify the cards to have manageable solutions so that the number of repetitions performed met the ability of the class. Teachers were also encouraged to create their own mental math problems based on their course content for that day or week.

##### Sedentary Control Condition

Students in the control condition engaged in their regular classroom math work seated at a desk.

#### Activity Dose

The activity dose consisted of either 5-, 10-, or 20-min of physical activity or sedentary math work.

### Primary Outcome Measures

#### Executive Functioning

To assess the three core executive functions of inhibition, switching, and updating, three tasks were chosen that have been commonly used in previous research to assess executive functioning in general (65) as well as to assess the effects of acute exercise on executive functioning (10). As described below, these three tasks were administered prior to (pre-test) and following the experimental manipulations (post-test) and were performed in the same order at each assessment beginning with the Stroop task (inhibition), followed by the Trail Making Test (switching), and then the Forward Working Memory test (updating).

#### Inhibition

The congruent and incongruent versions of the Stroop task (66) were used to assess inhibition. Participants performed the congruent version for 1-min followed by the incongruent version for 5-min. Both versions consisted of lists of words printed on laminated sheets of paper. In the congruent version, the words and the print ink color were matched (e.g., ink color was *green* and the word text read *green*), and participants were asked to read the word aloud (i.e., green). In the incongruent version, the words and print ink color were mismatched (e.g., ink color was *yellow* and the word text read *green*), and participants were asked to say aloud the ink color they saw (i.e., yellow) without reading the word text. In both versions, children were asked to try to respond as fast and accurately as possible. If an error was made it was recorded by the research assistant. Children were often unaware of the errors they made but if they corrected their response it was still recorded as an error by the research assistant. A total Stroop task performance score (i.e., Stroop accuracy) was computed by subtracting the amount of errors made on each version from the amount of words completed on each version, and then summing those values [i.e., Stroop task performance score = (congruent words completed—congruent errors made) + (incongruent words completed—incongruent errors made)]. This calculation was conducted separately for the pre- and post-test assessments and has been used in previous research when investigating the acute effects of physical activity on executive functioning (67). Higher scores indicate better performance on the Stroop task.

#### Switching

The Trail Making Test [TMT; (68)] was used to assess switching as it is a valid and appropriate measure for children (69). The TMT consists of two parts, Part A and Part B. Part A requires participants to connect number sequences, whereas Part B requires participants to alternate between number and letter sequences. In both versions, participants are required to connect the sequences in order, as fast and accurately as possible, without lifting their pencil or turning the paper. If an error was committed (i.e., connected the wrong sequence) or a pencil lift was made, it was recorded by the research assistant under “total errors.” A total TMT performance score was computed by adding the total errors committed to the time (in seconds) it took the participants to complete each version, and then summing those values [i.e., TMT performance score = (total time Part A + total errors Part A) + (total time Part B + total errors Part B)]. This calculation was conducted separately for the pre- and post-test assessments and has been used in previous research when investigating the acute effects of physical activity on executive functioning (67). Lower scores indicate better performance on the TMT.

#### Updating

The Forward Memory Span (FMS) test from the Leiter International Performance Scale–3rd Edition was used to assess updating as it is a valid and appropriate measure for children (70). The FMS test is a non-verbal assessment whereby the participant is presented with pictures of objects shown in a grid pattern (e.g., a 3 × 3 grid). First, the researcher points to multiple pictures (e.g., 4 pictures) in a predetermined order and then the participant is required to copy the same order. The grid pattern and number of pictures gradually increases as the test progresses. An error is recorded if the participant points to any of the pictures in the incorrect order. The test is terminated when six errors are committed or if the participant advances to the last sequence. A total FMS performance score was computed by summing the correct number of sequences performed, with a maximum score of 28. Higher scores indicate better performance on the FMS test.

#### Executive Functioning Composite Score

A single measure of executive functioning was calculated using standardized scores from each of the three core performance measures mentioned above. Specifically, standardized z-scores were calculated separately based on the total performance scores for the Stroop task, TMT, and FMS test at both assessments (i.e., at pre- and post-manipulation). However, the TMT z-scores were then multiplied by −1 so that TMT values were consistent with the Stroop task and FMS test values (i.e., positive values reflect better performance and negative values reflect poorer performance). The standardized scores were then summed to obtain separate composite scores for the pre- and post-test assessments, with higher values reflecting greater overall executive functioning performance.

### Secondary Outcome Measures

#### Mood

Positive mood was assessed using a modified version of the Profile of Mood States (71) that is suitable for children in response to physical exercise (72). Mood was assessed prior to pre- and post-test executive function assessments using 6-items from the positive affect subscale. Participants were asked to rate their current feeling state in response to each item on a scale ranging from 1 (*Not at All*) to 5 (*Extremely*). Positive mood items included *Active, Awake, Energetic, Excited, Friendly*, and *Happy*. A total positive mood score was computed by averaging the 6-items at each assessment. Internal consistency at each assessment was acceptable (Cronbach's α's > 0.79).

#### Motivation

Motivation for performing the executive function tests was assessed prior to the pre- and post-test executive function assessments using the effort and importance subscale from the Intrinsic Motivation Inventory (73). The effort and importance subscale has been successfully used with children in past research examining the effects of acute exercise on motivation and executive functioning (67, 74). The subscale contains 5-items that are rated on a scale ranging from 1 (*Not at all true*) to 7 (*Very true*). Each item was prefaced with the following stem “*For the brain games I'm about to do*.” An example item is: “*I am going to put a lot of effort into these brain games*.” A total motivation score was computed by averaging the 5-items at each assessment. Internal consistency at each assessment was good (α's > 0.89).

#### Task Self-Efficacy

Self-efficacy to perform the post-test executive function assessments was assessed using a four-item scale adhering to recommendations by Bandura (75) for assessing self-efficacy. This scale has also been successfully used with children in past research examining the effects of acute exercise on self-efficacy and executive functioning (67, 74). Each item was prefaced with the stem “*For the brain games I am about to do, I am confident I can perform…*.” The individual items represented gradations of performance that were relative to the participant's performance on the pre-test executive function assessments. They were (1) “*Almost as good as last time*,” (2) “*As good as last time*,” (3) “*A little better than last time*,” and (4) “*A lot better than last time*.” Participants rated their confidence (i.e., self-efficacy) for each item using an 11-point scale ranging from 0 (*Not at all Confident*) to 10 (*Completely Confident*). The task self-efficacy score was calculated by averaging the items. Internal consistency of the scale was acceptable (α = 0.79).

### Physical Fitness

Assessments of aerobic and musculoskeletal fitness were conducted by trained research assistants during the baseline data collection period for the larger intervention study within the school gymnasium.

#### Aerobic Fitness

The Leger 20-m Shuttle Run (SR) test was used to represent participants' aerobic fitness levels (76, 77), which is a valid and commonly used field-based measure of aerobic fitness in children (78, 79). The SR test involves running back and forth between two lines set 20 meters apart while maintaining a set pace with an audio signal that increases in difficulty over time (i.e., the signals become shorter). The test is terminated when a participant is unable to maintain the set pace for two consecutive audio signals. The number of laps completed served as the outcome of aerobic fitness. The higher number of laps completed represents better performance on the SR test.

Participants also rated their perceived exertion (RPE) using Borg's (80) CR-10 scale following the SR test. This was done to determine the extent to which they exerted their maximum physical effort on the test.

#### Musculoskeletal Fitness

Standing long jump (SLJ) was used to represent participants' musculoskeletal fitness, which is a common and valid field-based measure of musculoskeletal fitness in children (78). To perform a SLJ, participants were instructed to jump as far as they could using a 2-foot takeoff and a 2-foot landing from behind a marked line. Distance was measured to the nearest centimeter from the back of the closest heel to the line. Three attempts were made, with the longest distance used as the SLJ performance outcome.

### Data Analysis

All statistical analyses were conducted using SPSS 25 (81). Descriptive statistics were computed for all study variables. Chi-square tests and separate one-way analyses of variance (ANOVAs) were computed to assess differences in means between the physical activity and control conditions for demographic, anthropometric, and physical fitness scores. Separate 2 (activity type) × 3 (activity dose: 5- vs. 10- vs. 20-min) × 2 (time) repeated measures ANOVAs were computed to assess differences in means between conditions for the primary and secondary outcomes. However, as task self-efficacy was only assessed once (i.e., prior to the post-test executive function assessments), a 2 (activity type) × 3 (activity dose) univariate ANOVA was computed to assess differences in means between conditions for task self-efficacy.

A series of three separate, *post-hoc* exploratory, 2 (activity type) × 2 (time) repeated measures ANVOA analyses were conducted on the executive functioning composite scores. These exploratory analyses were primarily conducted due to the low number of participants (*n* = 12) in the traditional physical activity break condition. Specifically, we wanted to investigate (1) whether the academic physical activity break conditions (*n* = 37) and the sedentary control conditions (*n* = 67) differed on executive functioning after excluding the 12 participants from the traditional physical activity conditions, (2) whether the two physical activity break conditions differed on executive functioning, and (3) whether the traditional physical activity break condition and sedentary control condition differed on executive functioning.

Significant interactions were decomposed and evaluated using paired *t*-tests by comparing group means. Effect sizes for the one-way ANOVAs are reported as Cohen's *d* (82) and the values for small, medium and large are 0.20, 0.50, and 0.80, respectively. Effect sizes for the repeated measure ANOVAs and univariate ANOVAs are reported as partial eta squared ($\eta_{\text{p}}^{2}$) and the values for small, medium, and large are 0.01, 0.06, and 0.14, respectively.

**Figure 3:**
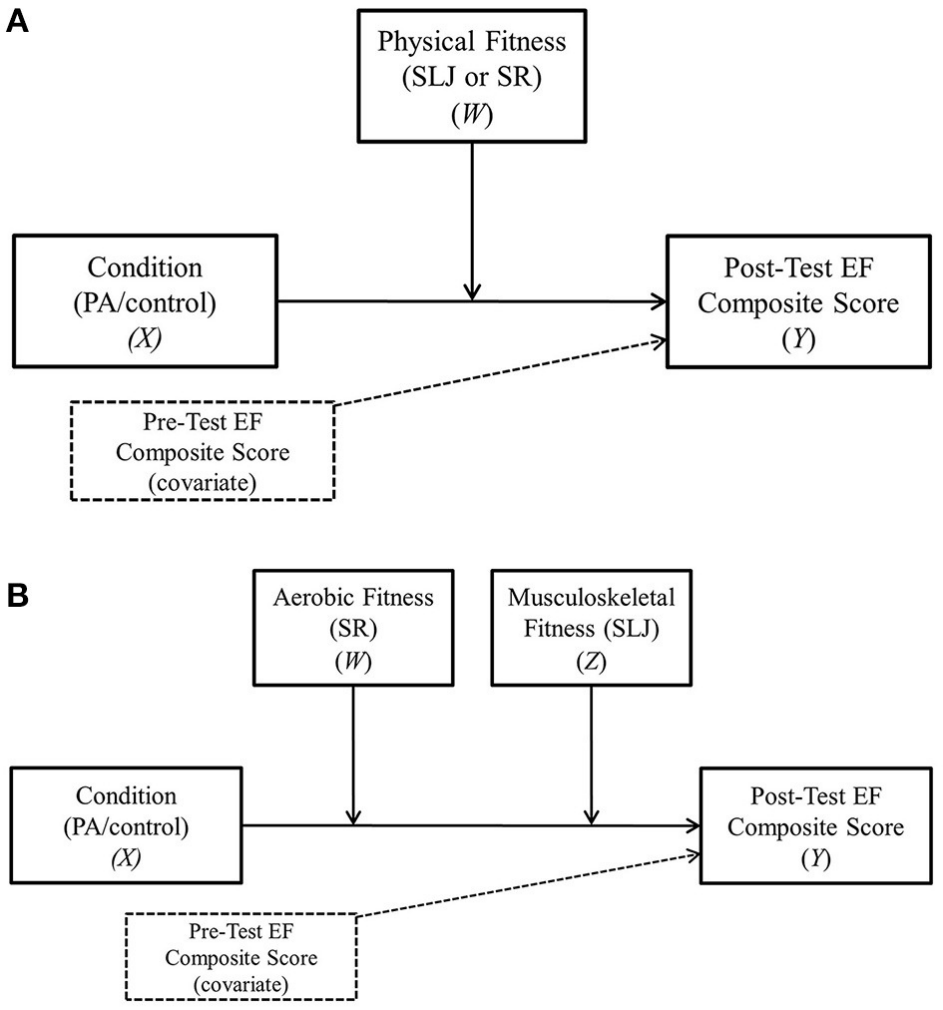
**(A)** Acute physical activity—executive functioning single moderation model. **(B)** Acute physical activity—executive functioning additive moderation model. SLJ, standing long jump; SR, shuttle run; EF, executive functioning.

Tests for single moderation were assessed using Model 1 in the PROCESS v3.5 software macro for SPSS (63). As recommended by Hayes and Scharkow (83), bias-corrected bootstrap procedures were computed utilizing 10,000 simulations. In each of the single moderation analyses (depicted in Figure 3A), the post-test executive function composite score served as the dependent variable (covarying for the pre-test executive function composite score), with experimental condition (physical activity vs. control) specified as the independent variable and aerobic or musculoskeletal physical fitness (i.e., SLJ distance or SR laps) as the moderator. The additive moderation analysis was conducted using Model 2 in the PROCESS software macro for SPSS (63) and bias-corrected bootstrap procedures utilizing 10,000 simulations were computed. In the additive moderation analysis (depicted in Figure 3B), the post-test executive function composite score served as the dependent variable (covarying for the pre-test executive function composite score), with experimental condition (physical activity vs. control) specified as the independent variable and both physical fitness outcomes (i.e., SLJ distance and SR laps) as the moderators. Significant (*p* < 0.05) moderation and conditional effects are indicated by a confidence interval that does not include zero. As recommended by Hayes (A. Hayes, personal communication, July 18, 2018), interactions that were significant at *p* < 0.10 were probed using either the Johnson-Neyman technique (single moderation) or by exploring the conditional effects of the moderators at the 16th, 50th, and 84th percentiles (additive moderation) as the Johnson-Neyman technique is not programmed in PROCESS when probing interactions in additive moderation analyses.

## Results

### Participant Characteristics

Descriptive statistics, chi-square, ANOVA summaries, and effect sizes for demographic, anthropometric, and physical fitness scores are shown, by group, in Table 1. Analyses revealed no significant differences (*p* > 0.05) between the sedentary control and physical activity break conditions. The only exception was that height values were significantly larger in the physical activity break condition when compared to the sedentary control condition (*p* = 0.03, *d* = 0.43).

**Table 1:** Demographic, anthropometric, and physical fitness scores by group.

	**Sedentary control *n* = 67**	**Physical activity break *n =* 49**			
	***M* (SD)**	***M* (SD)**	***F***	***p***	***d***
Age	12.22 (1.02)	12.51 (0.96)	2.36	0.13	0.30
BMI	19.69 (4.89)	19.70 (4.59)	0.00	0.99	0.002
Height (cm)	152.09 (11.28)	156.13 (7.15)	4.84	0.03	0.43
Weight (kg)	45.86 (13.55)	48.45 (13.44)	1.03	0.31	0.19
SLJ (cm)	142.24 (26.23)	151.08 (27.25)	3.05	0.08	0.33
SR final stage	5.08 (2.22)	5.15 (1.92)	0.03	0.86	0.03
SR laps completed	38.08 (20.02)	39.02 (18.21)	0.86	0.80	0.05
SR RPE	7.17 (2.13)	7.13 (1.84)	0.01	0.91	0.02
			***x*** ^2^	***p***	***V***
Girls/Boys	34/33	24/25	0.03	0.85	0.02
White/Other	48/19	36/13	0.05	0.83	0.02

*M, mean; SD, standard deviation; BMI, body mass index; cm, centimeters; kg, kilograms; SLJ, standing long jump; SR, shuttle run; d, Cohen's d; V, Cramer's V*.

### Primary Analyses

Descriptive statistics, ANOVA summaries, and effect sizes for the Stroop task, the TMT, the FMS test, and the executive functioning composite score are shown, by group, in Table 2.

**Table 2:** Executive functioning, mood, motivation, and task self-efficacy scores by group.

	**Sedentary control**				**Physical activity break**						
	**Pre**		**Post**		**Pre**		**Post**		***F***	***p***	**$\eta_{\text{p}}^{2}$**
	***M***	***SD***	***M***	***SD***	***M***	***SD***	***M***	***SD***			
Stroop task	297.45	(54.25)	326.88	(65.15)	299.04	(54.27)	362.33	(61.00)	25.19^a^	<0.001	0.19
TMT	128.70	(42.09)	126.89	(44.07)	125.53	(37.46)	115.21	(31.45)	1.22^a^	0.27	0.01
FMS test	22.57	(2.43)	22.58	(2.05)	21.96	(2.23)	23.04	(2.48)	6.42^a^	0.01	0.06
EF composite score	0.99	(2.45)	−0.42	(2.30)	−0.13	(2.23)	0.57	(2.21)	16.24^a^	<0.001	0.13
Mood	3.64	(0.57)	3.46	(0.66)	3.37	(0.60)	3.92	(0.71)	42.19^a^	<0.001	0.28
Motivation	5.72	(0.92)	5.77	(0.96)	5.61	(0.99)	5.86	(0.98)	5.61^a^	0.02	0.05
Self-efficacy	–	–	5.70	(1.82)	–	–	7.20	(1.87)	15.76^b^	<0.001	0.08

*M, mean; SD, standard deviation; TMT, trail making test; FMS, forward memory span; EF, executive functioning; $\eta_{\text{p}}^{2}$, partial eta squared*.

a *F-values represent the time by activity type interaction from the 2 × 3 repeated measures ANOVAs*.

b *F-value represents the main effect for activity type from the 2 × 3 univariate ANOVA*.

#### Executive Functioning Composite Score

**Figure 4:**
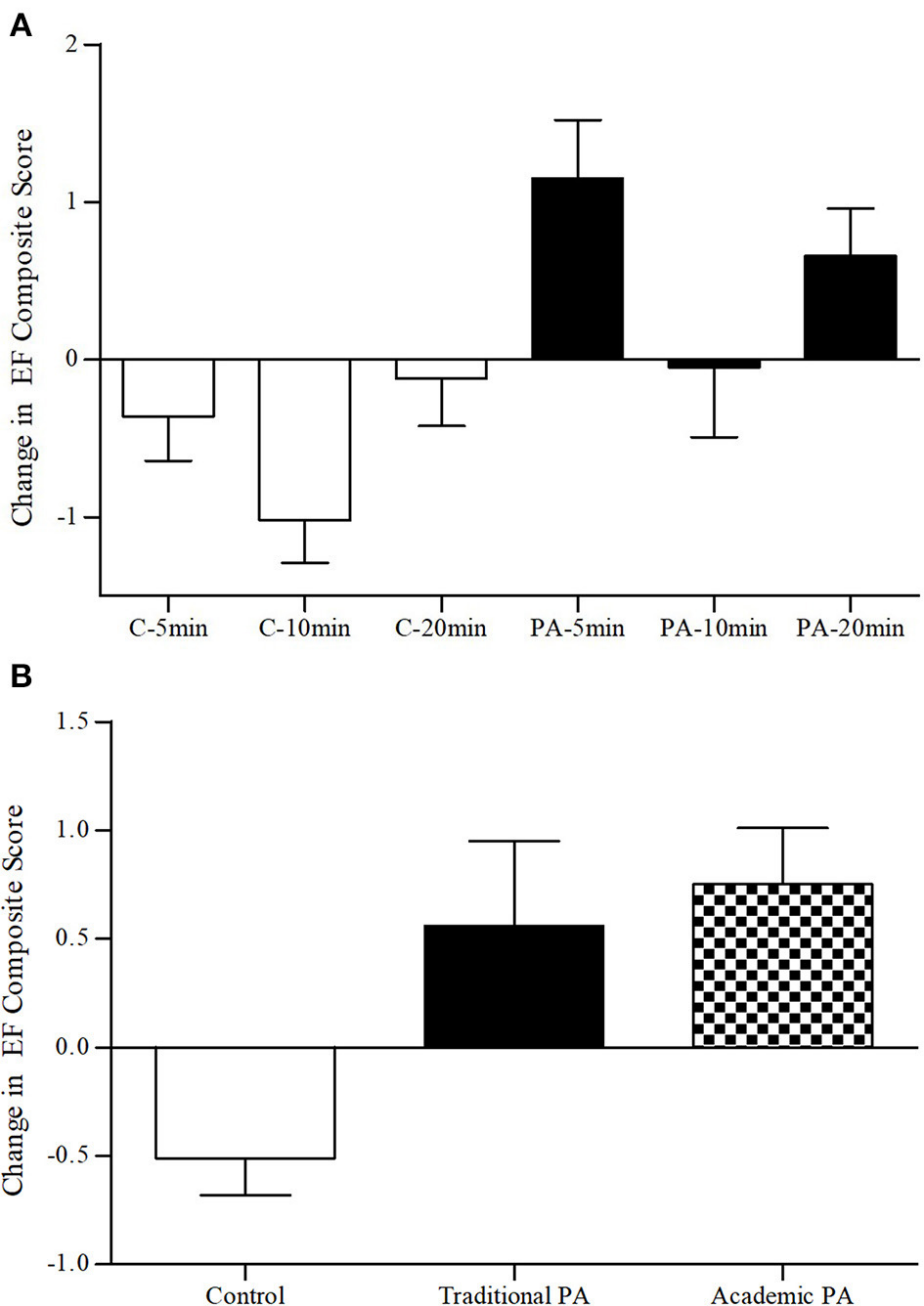
**(A)** Change in executive functioning composite scores by activity type and dose. **(B)** Change in executive functioning composite scores by activity type. EF, executive functioning; C, sedentary control; PA, physical activity break; min, minutes. Error bars represent SE of the mean.

Results of the 2 × 3 × 2 repeated measures ANOVA for the executive functioning composite score revealed a non-significant main effect for time (*p* = 0.76, $\eta_{\text{p}}^{2}$ = 0.001). However, the time by activity type interaction (*p* < 0.001, $\eta_{\text{p}}^{2}$ = 0.13) and the time by activity dose interaction (*p* = 0.02, $\eta_{\text{p}}^{2}$ = 0.07) were significant. The time by activity type by activity dose interaction was not significant (*p* = 0.50, $\eta_{\text{p}}^{2}$ = 0.01). Specifically, as depicted in Figure 4A, the executive functioning composite scores increased significantly among participants in the 5-min (*p* = 0.01, *d* = 0.51) and 20-min (*p* = 0.04, *d* = 0.31) physical activity break conditions, they remained relatively stable in the 10-min physical activity break (*p* = 0.91, *d* = 0.02) and the 20-min sedentary control (*p* = 0.69, *d* = 0.04) conditions, and decreased in the 5-min sedentary control (*p* = 0.21, *d* = 0.14) and 10-min sedentary control (*p* = 0.001, *d* = 0.58) conditions.

Results of the first, *post-hoc*, 2 × 2 repeated measures ANOVA for the change in executive function composite scores between the academic physical activity break conditions and the sedentary control conditions revealed no main effect for time (*p* = 0.43, $\eta_{\text{p}}^{2}$ = 0.01), however the time by activity type interaction was significant (*p* < 0.001, $\eta_{\text{p}}^{2}$ = 0.15). Results of the second 2 × 2 repeated measures ANOVA for the change in executive function composite scores between the academic physical activity break conditions and the traditional physical activity break conditions revealed a significant main effect for time (*p* = 0.012, $\eta_{\text{p}}^{2}$ = 0.12), however the time by activity type interaction was not significant (*p* = 0.71, $\eta_{\text{p}}^{2}$ = 0.01). Results of the third 2 × 2 repeated measures ANOVA for the change in executive function composite scores between the traditional physical activity break conditions and the sedentary control conditions revealed no main effect for time (*p* = 0.91, $\eta_{\text{p}}^{2}$ = 0.00), however the time by activity type interaction was significant (*p* = 0.01, $\eta_{\text{p}}^{2}$ = 0.08). Specifically, as depicted in Figure 4B, executive functioning composite scores increased significantly among participants in the traditional physical activity conditions (*p* =.006, *d* = 0.22) and academic physical activity break conditions (*p* = 0.003, *d* = 0.35), whereas they decreased among participants in the sedentary control conditions (*p* = 0.18, *d* = 0.22).

#### Inhibition

**Figure 5:**
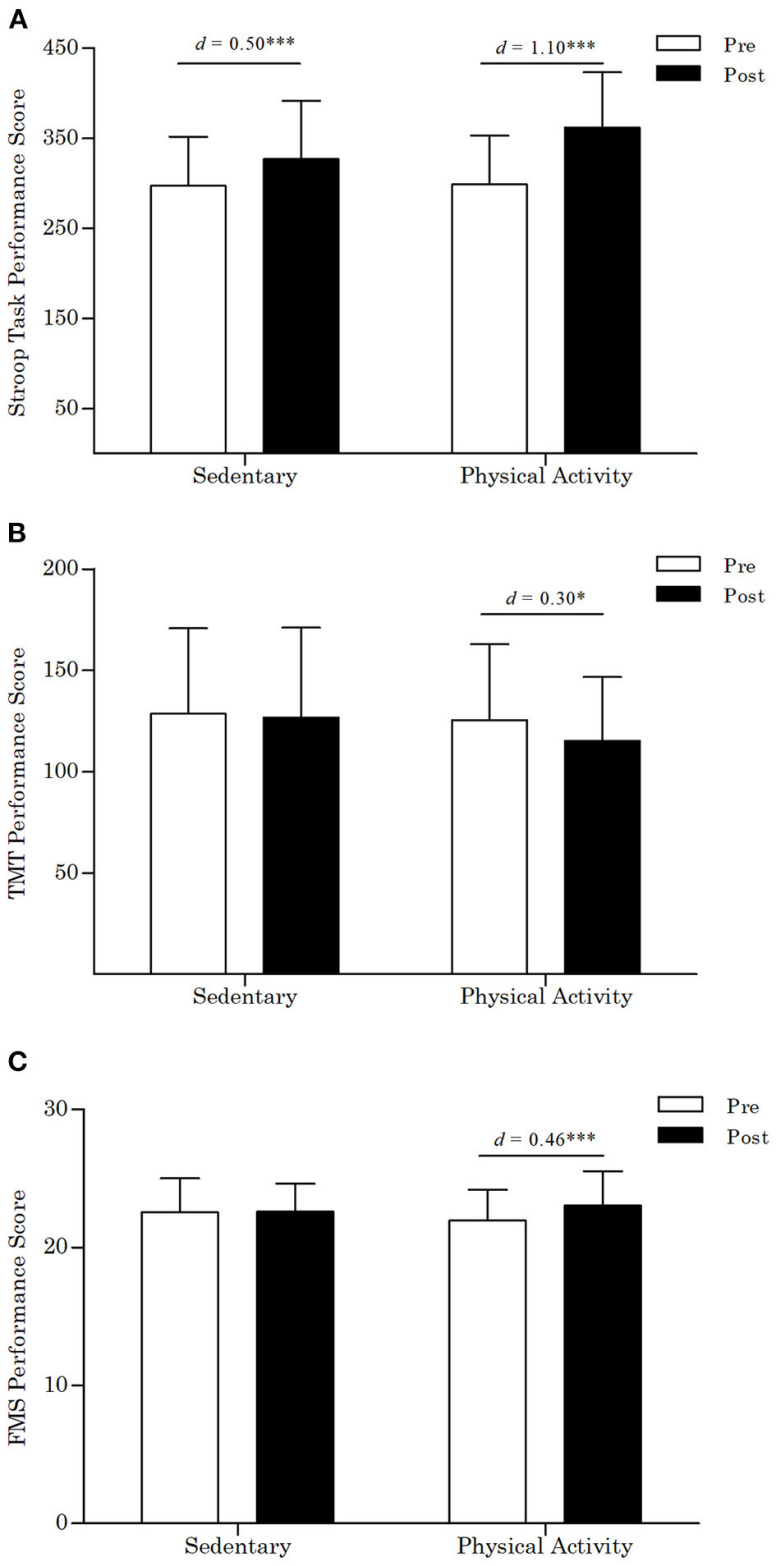
**(A)** Stroop task performance scores by condition. **(B)** Trail making test performance scores by condition. TMT, trail making test. Lower scores indicate better performance on the TMT. **(C)** Forward memory span test scores by condition. FMS, forward memory span test. *d*, Cohen's *d*, ***p < 0.001, *p < 0.05. Error bars represent SD of the mean.

Results of the 2 × 3 × 2 repeated measures ANOVA for the Stroop task performance score revealed a significant main effect for time (*p* < 0.001, $\eta_{\text{p}}^{2}$ = 0.19) and a significant time by activity type interaction (*p* < 0.001, $\eta_{\text{p}}^{2}$ = 0.19). However, the time by activity dose interaction (*p* = 0.13, $\eta_{\text{p}}^{2}$ = 0.04) and the time by activity type by activity dose interaction (*p* = 0.73, $\eta_{\text{p}}^{2}$ = 0.01) were not significant. Specifically, as depicted in Figure 5A, Stroop task performance scores significantly increased to a greater extent among participants in the physical activity conditions (*p* < 0.001, *d* = 1.10) when compared to participants in the sedentary control conditions (*p* < 0.001, *d* = 0.50).

#### Switching

Results of the 2 × 3 × 2 repeated measures ANOVA for the TMT performance score revealed no significant findings for the main effect for time (*p* = 0.08, $\eta_{\text{p}}^{2}$ = 0.03), the time by activity type interaction, (*p* = 0.27, $\eta_{\text{p}}^{2}$ = 0.01), the time by activity dose interaction (*p* = 0.26, $\eta_{\text{p}}^{2}$ = 0.02), and the time by activity type by activity dose interaction (*p* = 0.59, $\eta_{\text{p}}^{2}$ = 0.01). Although the time by activity type interaction was not significant, *post-hoc* exploratory analyses showed that TMT performance scores decreased significantly (i.e., participants performed better) among participants in the physical activity conditions (*p* = 0.03, *d* = 0.30) whereas they remained relatively stable among participants in the sedentary control conditions (*p* = 0.58, *d* = 0.04), as depicted in Figure 5B.

#### Updating

Results of the 2 × 3 × 2 repeated measures ANOVA for the FMS test performance score revealed a significant main effect for time (*p* = 0.01, $\eta_{\text{p}}^{2}$ = 0.06) and a significant time by activity type interaction (*p* = 0.01, $\eta_{\text{p}}^{2}$ = 0.06). However, the time by activity dose interaction (*p* = 0.26, $\eta_{\text{p}}^{2}$ = 0.02) and the time by activity type by activity dose interaction (*p* = 0.70, $\eta_{\text{p}}^{2}$ = 0.01) were not significant. Specifically, as depicted in Figure 5C, FMS test scores increased significantly among participants in the physical activity conditions (*p* < 0.001, *d* = 0.46) whereas they remained relatively stable among participants in the sedentary control conditions (*p* = 0.95, *d* = 0.01).

### Secondary Analyses

#### Mood

Results of the 2 × 3 × 2 repeated measures ANOVA for the positive mood scores revealed a significant main effect for time (*p* = 0.001, $\eta_{\text{p}}^{2}$ = 0.10) and a significant time by activity type interaction (*p* < 0.001, $\eta_{\text{p}}^{2}$ = 0.28). However, the time by activity dose interaction (*p* = 0.13, $\eta_{\text{p}}^{2}$ = 0.04) and the time by activity type by activity dose interaction (*p* = 0.87, $\eta_{\text{p}}^{2}$ = 0.003) were not significant. Specifically, as seen in Table 2, mood scores increased significantly among participants in the physical activity condition (*p* < 0.001, *d* = 0.83) whereas they decreased among participants in the sedentary control condition (*p* = 0.01, *d* = 0.28).

#### Motivation

Results of the 2 × 3 × 2 repeated measures ANOVA for the motivation scores revealed a significant main effect for time (*p* = 0.001, $\eta_{\text{p}}^{2}$ = 0.09) and a significant time by activity type interaction (*p* = 0.02, $\eta_{\text{p}}^{2}$ = 0.05). However, the time by activity dose interaction (*p* = 0.57, $\eta_{\text{p}}^{2}$ = 0.01) and the time by activity type by activity dose interaction (*p* = 0.62, $\eta_{\text{p}}^{2}$ = 0.01) were not significant. Specifically, as seen in Table 2, motivation scores increased significantly among participants in the physical activity condition (*p* < 0.001, *d* = 0.25) whereas they remained relatively stable among participants in the sedentary control condition (*p* = 0.39, *d* = 0.05).

#### Task Self-Efficacy

**Figure 6:**
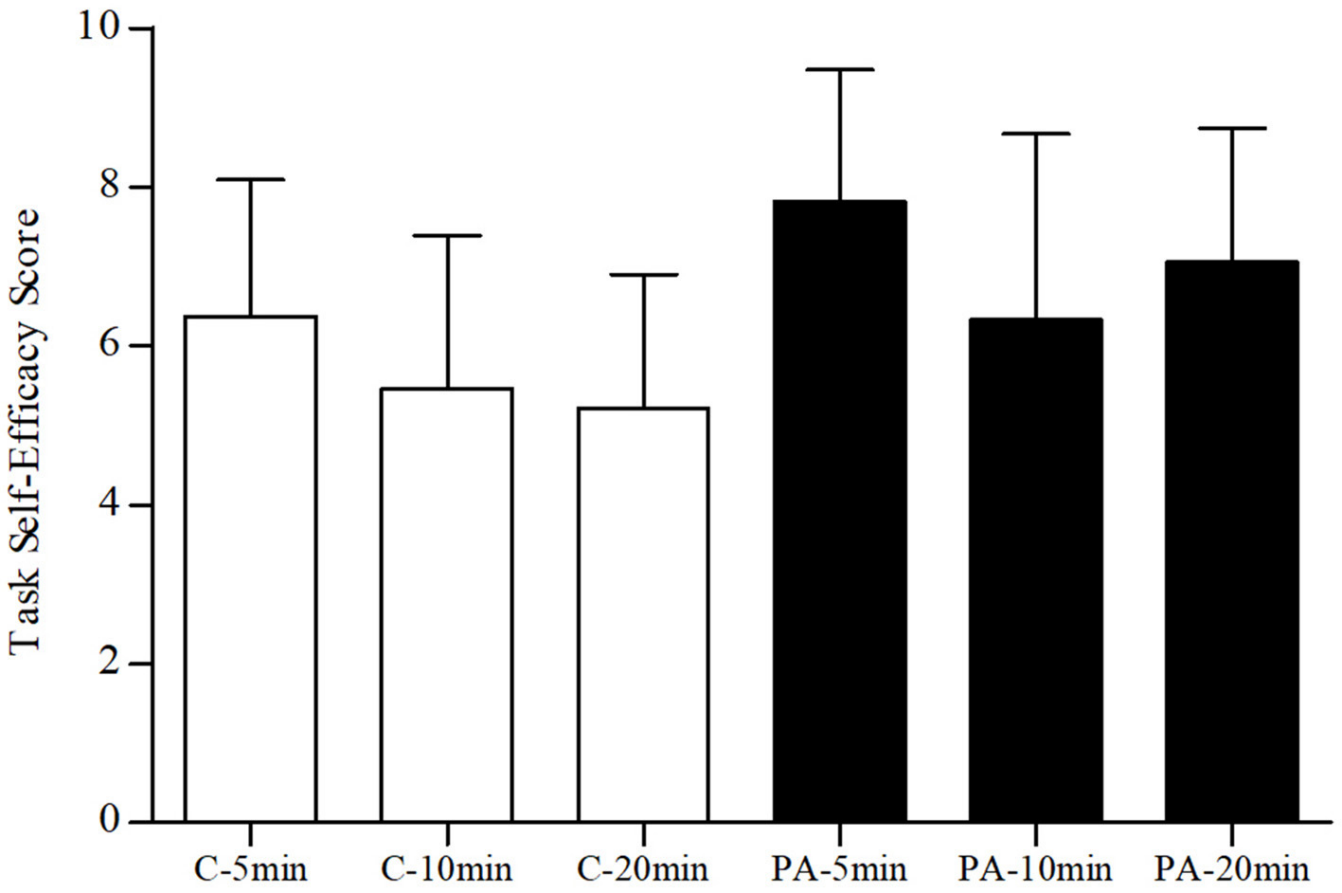
Task self-efficacy scores by condition. C, sedentary control; PA, physical activity break; min, minutes. Error bars represent SD of the mean.

Results of the 2 × 3 univariate ANOVA for the self-efficacy scores revealed significant main effects for activity type (*p* < 0.001, $\eta_{\text{p}}^{2}$ = 0.13) and activity dose (*p* = 0.01, $\eta_{\text{p}}^{2}$ = 0.08). The activity type by activity dose interaction was not significant (*p* = 0.54, $\eta_{\text{p}}^{2}$ = 0.01). As seen in Table 2, on average, self-efficacy scores were higher among participants in the physical activity break conditions when compared to the sedentary control conditions. LSD *post-hoc* analyses revealed that participants in the 5-min conditions reported significantly higher self-efficacy scores when compared to the 10-min (*p* = 0.002) and 20-min (*p* = 0.02) conditions, whereas the 10- and 20-min condition scores were not significantly different from one another (*p* = 0.62). However, as seen in Figure 6, higher mean scores for the 5-min break conditions were primarily driven by the 5-min physical activity break condition.

### Moderation Analyses

Results of the first single moderation analysis (depicted in Figure 3A) revealed no significant main effects for condition (95% C.I. = −1.16, 1.17, *p* = 0.99) and SR laps completed (95% C.I. = −0.03, 0.01, *p* = 0.33) on the post-test executive functioning composite score. However, the condition by SR laps completed interaction was significant (95% C.I. = 0.01–0.05, *p* = 0.04), covarying for the pre-test executive functioning composite score. When probing this interaction, results from the Johnson-Neyman technique revealed a conditional effect (*p* < 0.05) at 25.40 laps and above on the SR test. This indicates, among participants in the physical activity break condition, those who completed 25.40 laps or above (i.e., 26 laps or above) experienced greater increases in executive functioning on the post-test when compared to participants who completed 25 laps or fewer. This was also a linear relationship as the conditional effect gradually increased alongside aerobic fitness levels (i.e., effect for 25.40 laps = 0.71 and the effect for 95.00 laps = 2.63). In other words, as aerobic fitness levels increased, executive functioning performance also increased whereby the most aerobically fit children showed the greatest improvements in executive functioning performance following the physical activity break.

Results of the second single moderation analysis (depicted in Figure 3A) revealed no significant main effects for condition (95% C.I. = −0.68, 5.07, *p* = 0.13) and SLJ distance (95% C.I. = −0.02, 0.01, *p* = 0.68), as well as a non-significant interaction (95% C.I. = −0.03, 0.01, *p* = 0.49), covarying for the pre-test executive functioning composite score.

Results of the additive moderation analysis (depicted in Figure 3B) revealed no significant main effects for condition (95% C.I. = −0.55, 5.37, *p* = 0.11), SR laps completed (95% C.I. = −0.03, 0.01, *p* = 0.35), and SLJ distance (95% C.I. = −0.01, 0.02, *p* = 0.78). However, the condition by SR laps interaction was significant (95% C.I. = 0.01, 0.08, *p* = 0.01), covarying for the pre-test executive functioning composite score. The condition by SLJ distance interaction was not significant (95% C.I. = 0.04, 0.004, *p* = 0.09), covarying for the pre-test executive functioning composite score. As both interactions were below *p* = 0.10, the conditional effects were examined. Findings revealed that the conditional effect for low levels of aerobic fitness (i.e., at the 16th percentile for SR laps) was not significant (*p* > 0.05) across each level of musculoskeletal fitness (i.e., at the 16th, 50th, and 84th percentiles for SLJ distance). However, as aerobic fitness increased (i.e., at the 50th and 84th percentiles), the effect of musculoskeletal fitness became significant across each level. These results suggest, among participants in the physical activity break conditions, that both aerobic and musculoskeletal fitness influence executive functioning performance but only beginning at moderate levels of aerobic fitness.

## Discussion

The present study investigated the acute effects of a classroom-based, teacher-delivered, physical activity break during math class on executive functioning in 11–14-year-old children when compared to sedentary seated classroom work. We also investigated the potential independent (see Figure 3A) and combined (see Figure 3B) moderating effects of both aerobic and musculoskeletal fitness on the acute physical activity—executive functioning relationship. Findings from the present study support previous literature illustrating how acute physical activity and physical fitness can promote aspects of executive functioning and cognition related to academic achievement and school readiness. Our findings also build on previous literature by demonstrating how aspects of physical fitness (cardiorespiratory and musculoskeletal) moderate the acute physical activity—executive functioning relationship. Together, these findings further highlight the importance of incorporating physical activity breaks throughout the school day and especially within a classroom setting.

### Classroom-Based Physical Activity and Executive Functioning

Consistent with our hypotheses, children who participated in the physical activity breaks showed improvements in executive functioning across measures of inhibition, switching, and updating when compared to children in the secondary control conditions (depicted in Figures 4, 5). These findings support previous research showing the efficacy for relatively short, acute, classroom-based physical activity breaks on aspects of executive functioning in children (19). Our findings also suggest that the 5-, 10-, and 20-min physical activity breaks uniformly, and positively, impacted executive functioning. These findings build on limited research examining the effects of different doses of acute classroom-based physical activity on executive functioning within a single study (43).

The lack of findings with regard to dose also have important practical implications as they suggest teachers can not only implement relatively short physical activity breaks (i.e., 5–10 min) and reap subsequent benefits on executive functions related to learning, but they also have implications for the implementation of school-based DPA initiatives across the school day. Specifically, findings from the present study suggest teachers can strategize how they implement DPA over the course of a school day while still reaping the acute benefits of physical activity breaks on executive functioning. For example, depending on the age of the students, subject, or lesson plans for that day, teachers could implement multiple 5- or 10-min breaks across the school day during instructional time to meet the 20-min DPA guideline in Ontario (as discussed in the introduction). Given previous research suggests teachers often struggle to implement 20 min of sustained DPA (23, 25) despite additional support [e.g., (84)], and when they do implement DPA the bouts generally last 5–10 min (25), our findings suggest that utilizing short physical activity breaks over the school day may provide an alternative strategy to implement DPA that could also have positive effects on physical, cognitive, and mental health outcomes over the course of a school year.

We also hypothesized that the academic physical activity breaks would lead to greater changes in executive functioning when compared to the traditional physical activity breaks and the sedentary control conditions. Unfortunately, we encountered an unexpected setback after baseline testing which limited data collection and access to participants at one school. This resulted in substantially fewer participants in the traditional physical activity break conditions (*n* = 12) when compared to the academic physical activity break conditions (*n* = 37). Although *post-hoc* analyses suggested these conditions did not differ significantly on changes in executive functioning and both conditions significantly outperformed the control condition (depicted in Figure 4B), our findings are limited when comparing the two types of physical activity conditions. The lack of differences between different types of activities requiring varying degrees of cognitive engagement, when compared to sedentary or control conditions, has also been observed previously (41). From a practical perspective, these results simply suggest that both types of activity breaks can be advantageous when compared to sedentary classroom work and that teachers can preserve instructional time by incorporating academic material within the physical activity breaks.

Results from the secondary psychological outcomes also provide insight to the positive carryover effects of classroom-based physical activity breaks (also see Table 2). That is, following the physical activity break, students reported higher levels of positive mood in general (e.g., happy and energetic), were more intrinsically motivated to invest effort into the executive function tests and felt that it was important to do so, and they also felt more confident (i.e., higher self-efficacy) in their ability to do well on the post-test executive function assessments when compared to the pre-test assessments. Independently, the findings with regards to mood, motivation, and self-efficacy are important and represent an underappreciated area of investigation with regards to potential psychological mechanisms for the acute effects of physical activity on executive functioning, including classroom-based physical activity breaks (85). However, collectively, the findings are that much more interesting. For example, it is likely that positive mood (or affective valence) following acute physical activity triggers a cascade of psychological responses that not only influence one's motivation and confidence to perform demanding cognitive tasks that involve executive functions but, in turn, may also help to buffer other maladaptive psychological responses (i.e., anxiety and doubt) that are often negatively associated with demanding cognitive tasks. Indeed, increases in mood, arousal, and affect are associated with both physiological and psychological processes related to task performance including motivation, self-efficacy, and aspects of executive functioning [for examples see (36, 86–92)]. Findings from the present study also suggest children felt the most confident in their ability to perform the post-test executive function assessments following the 5-min physical activity break (see Figure 6) which lends additional support to the rationale of breaking up DPA into shorter bouts over the school day and across subject areas.

### Moderating Effects of Physical Fitness

Levels of aerobic and musculoskeletal fitness were both found to influence the acute physical activity—executive functioning relationship (depicted in Figures 3A,B). With regards to aerobic fitness, we found a moderating effect for performance on the shuttle run (SR) test whereby children in the physical activity conditions who completed 25.40 laps (i.e., 26 laps) or higher performed significantly better on the post-test executive function assessments when compared to children who completed <25 laps. In addition, a linear relationship was observed in the conditional effect output from the Johnson-Neyman technique indicating executive functioning performance increased as aerobic fitness increased. These findings support the potential influence of high levels of aerobic fitness following acute physical activity on executive functioning, however additional research is needed given inconsistencies within the literature (42, 55, 56, 58–62).

With regards to musculoskeletal fitness [i.e., standing long jump (SLJ)], findings from the single moderation analysis were not significant. However, findings from the additive moderation analysis (depicted in Figure 3B) were significant and extend previous cross-sectional research on the relationship between aspects of physical fitness and executive functioning in children (53). Specifically, findings revealed that levels of both aerobic and musculoskeletal fitness interact and positively influence executive functioning following acute physical activity beginning at moderate levels of aerobic fitness (i.e., 50th percentile = 37 laps on the SR test) across levels of musculoskeletal fitness (i.e., 16th percentile = 118.76 cm on SLJ, 50th percentile = 145.00 cm, and 84th percentile = 173.24 cm). In other words, musculoskeletal fitness enhances executive functioning following acute physical activity to a greater extent among children with moderate levels of aerobic fitness whereas musculoskeletal fitness has no added benefit for those with low levels of aerobic fitness (which aligns with findings from the single moderation analysis of aerobic fitness in the sense that low levels of aerobic fitness had no influence on executive functioning following physical activity). Moreover, examination of the conditional effects output suggests the interacting effects of aerobic and musculoskeletal fitness on executive functioning are, on average, highest among those with higher levels of aerobic fitness (i.e., 84th percentile = 57.48 laps on SR test), again highlighting the importance of high levels of aerobic fitness. Collectively, these results provide preliminary insight on the moderating effects of both aerobic and musculoskeletal fitness (i.e., when included as continuous variables) when examining the acute physical activity—executive functioning relationship. Yet, future research is needed to replicate these results.

The interacting effects of aerobic and musculoskeletal fitness on executive functioning following acute physical activity provide important considerations for school based physical activity initiatives. Specifically, many initiatives' primary focus is to increase physical activity levels throughout the school day to meet recommended guidelines and help eradicate the physical inactivity crisis among children and youth [as discussed by (6)]. In addition, these initiatives discuss various ways to increase physical activity during the school day, during before/afterschool programs, and the importance of using health and physical education courses to educate students on the value of engaging in physical activity regularly and, ultimately, to learn to be physically active for a lifetime. However, findings from the present study suggest that targeting physical fitness levels should also be a focus alongside increasing physical activity among school-based physical activity initiatives. For instance, it may be advantageous to target aspects of physical fitness during physical education (in addition to educating students on how to increase their fitness and physical activity levels) and then target physical activity levels within the classroom. By doing so, physical education specialists can indirectly affect students' academic achievement and school readiness (through physical fitness) while teachers can also directly reap these additional benefits following classroom-based physical activity breaks, especially if the breaks contain academic content. Although our findings are preliminary and were among 11–14-year-old children, the interacting effects of physical fitness on the acute physical activity—executive functioning relationship likely apply to other age groups where the acute effects of physical activity have been previously observed (e.g., preschool, elementary and high school-aged children, and youth). This however requires future research for confirmation.

Given the SR test is one of the most widely used and valid field-based measures of aerobic fitness among children and youth (78), very large datasets have been amassed that present normative ranges across sexes and age groups (79). As seen in Table 1 from the present study, the average SR laps completed for the physical activity break condition was 39.02 and subsequent analyses within that condition indicated that boys completed 42.64 laps on average and girls completed 34.91 laps on average. As indicated in Table 3 from Tomkinson et al. (79), the 50th percentile range for boys aged 11–14-years-old is 36–48 laps and the 50th percentile range for girls aged 11–14-years-old is 28–29 laps. Using Table 3 and the quintile framework^1^ outlined in Tomkinson et al. [(79), *p*. 7], boys in the present study would be classified as having “moderate” levels of aerobic fitness (between 40th and 60th percentiles) whereas girls would be classified as having “high” levels of aerobic fitness (between 60th and 80th percentiles). However, findings from the aerobic fitness single moderation analysis in the present study indicated children in the physical activity condition who completed 25.40 laps and above (i.e., 26 laps and above) experienced greater increases on the post-test executive function assessments when compared to children who completed fewer than 25.40 laps. Again, using Table 3 and the quintile framework outlined in Tomkinson et al. (79), 25.40 laps fall within the 20th percentile and a “low” level of aerobic fitness for boys aged 11–14-years old whereas 25.40 laps fall within the 40th percentile and a “moderate” level of aerobic fitness for girls. Therefore, findings from the present study suggest that, for children aged 11–14-years old, boys with low levels of aerobic fitness (and above) and girls with moderate levels of aerobic fitness (and above) show greater improvements in executive functioning following acute physical activity when compared to their peers with lower levels of aerobic fitness.

The above findings could have important implications within a school setting whereby teachers and/or the physical education specialist(s) have their 11–14-year-old students complete the SR test at the beginning of the school year to establish baseline levels of aerobic fitness. Results of this test would help identify if any boys or girls scored lower in aerobic fitness (i.e., below “low” for boys and “moderate” for girls) whereby they may not reap the additional cognitive benefits of an acute classroom-based physical activity break. If any students fall below this aerobic fitness “threshold,” the physical education specialist may wish to target exercises, games, or sports that increase aerobic fitness early in the school year. As discussed by Tomkinson et al. (79), previous research suggests a 12-week aerobic training program in children (93) can lead to an increase in ~20 centile points and should move children who score “very low” or “low” to a higher level of aerobic fitness whereby they reap the cognitive benefits of acute physical activity. In addition, aerobic training over time should also improve various aspects of physical health among the majority of boys and girls within this age range based on criterion-referenced standards for the SR test (94, 95).

### Strengths, Limitations, and Future Directions

Findings from the present study provide several exciting avenues for future research, however there are several limitations that should be discussed. For example, although the study has high ecological validity as it was conducted in a classroom-setting, during math class, and teachers delivered the physical activity breaks to the entire class, we cannot be certain that each teacher delivered the physical activity breaks as instructed. Similarly, it is equally important to ensure that the physical activity breaks are delivered safely while maintaining a high degree of quality instruction. To achieve this, it may be worthwhile for teachers to receive a form of group exercise certification and/or work in conjunction with a physical education specialist.

Future research may wish to utilize pre-recorded videos designed for classroom settings, such as HOPSports Brain Breaks® (96), to ensure each student receives the same amount of physical activity. However, this may be challenging with regards to incorporating academic learning material alongside the physical activity breaks based on the subject, age, and lesson plan. As such, a mix of teacher-delivered and video-delivered activity breaks may be the most appropriate for the classroom setting. In addition to ensuring students receive the same dose and type of physical activity, it would be beneficial to assess whether students are actually engaging in the physical activity breaks. Following expert recommendations and previously established models, such as the CSPAP model [see (6)], is also encouraged in future work within school settings.

Another limitation was the small number of participants in the traditional physical activity break conditions due to complications we experienced with regards to our ability to collect data at one school. This also coincided with a relatively small sample within the 10-min physical activity break conditions as that school was assigned to the 10-min activity break conditions. While these setbacks were not anticipated, future research is encouraged to replicate the current study with equal cell sizes.

Although we observed some interesting differences between boys and girls with regards to the moderating effects of physical fitness, we recommend that future research investigates these relationships in greater detail. For instance, we found that boys with low levels of aerobic fitness (and above) and girls with moderate levels of aerobic fitness (and above) show greater improvements in executive functioning following acute physical activity when compared to their peers with lower levels of aerobic fitness. Given data suggests girls are generally less physically active and less physically fit when compared to boys and this gap tends to increase as they get older [e.g., (79, 97–99)], it would be worthwhile to investigate whether girls' executive functions (and academic performance) may benefit to a greater degree than boys over time through physical activity and physical fitness intervention research.

As previously mentioned in the introduction, research examining the effects of cognitively engaging physical activity and the integration of academic learning material alongside physical activity has been gaining significant interest. Various physiological and cognitive mechanisms as well as theoretical viewpoints have been proposed for the added benefit of this type of physical activity on executive functioning, cognition, and learning (19, 28, 29, 35, 36). However, with regards to classroom settings, Mavilidi et al. (19) recently proposed an innovative conceptual model and instructional method emphasizing the importance of considering which aspects of physical activity may be the most relevant (or similar) to the learning material. When the physical task and the cognitive or learning task are high on both integration and relevance [see Figure 1 on *p*. 7 from (19)], aspects of cognition and learning should be enhanced to a greater extent when compared to other combinations. For instance, traditional physical activity breaks during math class would be considered low on relevance and low on integration as the physical activity it not related to math. On the other hand, academic physical activity breaks that include solving math problems during math class would be considered low on relevance and high on integration since math is included during the break but the physical activity itself (i.e., jumping jacks, squats, running on the sport, etc.) is not related to the learning material. In turn, academic physical activity breaks should be more effective due to the higher degree of integration that facilitates cognition and learning. Mavilidi et al. (19) discuss several studies that fall under high integration/high relevance and future research is encouraged to utilize some of these strategies and to test this proposition both acutely and longitudinally across other age groups and subject areas.

## Conclusion

The present study has provided supporting evidence for the acute effects of classroom-based, teacher-delivered, physical activity breaks on executive functioning. The results suggest 5-, 10-, and 20-min physical activity breaks led to similar improvements in executive functioning when compared to sedentary conditions. Following the physical activity breaks, participants reported higher levels of positive mood, were more intrinsically motivated to invest effort into the executive function tests and felt that it was important to do so, and they also felt more confident in their ability to do well on the tests of executive functioning. Moderation analyses suggest both aerobic and musculoskeletal fitness impact the acute physical activity—executive functioning relationship, however additional research is necessary to replicate these moderating effects.

## Data Availability Statement

The raw data supporting the conclusions of this article will be made available by the authors, without undue reservation.

## Ethics Statement

The studies involving human participants were reviewed and approved by McMaster University Research Ethics Board and the Hamilton-Wentworth Catholic District School Board Research Ethics Committee. Written informed consent to participate in this study was provided by the participants' legal guardian/next of kin.

## Author Contributions

JG conducted the data analyses and drafted the initial version of the manuscript. JG and EB designed the study, coordinated and carried out participant recruitment, and data collection. BF assisted in data collection. JC supervised the design and execution of all phases of the study. All authors reviewed and approved the final manuscript.

## Conflict of Interest

The authors declare that the research was conducted in the absence of any commercial or financial relationships that could be construed as a potential conflict of interest.

## Publisher's Note

All claims expressed in this article are solely those of the authors and do not necessarily represent those of their affiliated organizations, or those of the publisher, the editors and the reviewers. Any product that may be evaluated in this article, or claim that may be made by its manufacturer, is not guaranteed or endorsed by the publisher.

## Footnotes

^1^Tomkinson et al.'s [(79), p. 7] quintile framework categorizes cardiorespiratory endurance levels as “very low” (below the 20th centile), “low” (between 20th and 40th centiles), “moderate” (between 40th and 60th centiles), “high” (between 60th and 80th centiles), and “very high” (above the 80th centile).
